# Inheritance of the duration of fertility in chickens and its correlation with laying performance

**DOI:** 10.1186/s12711-022-00733-7

**Published:** 2022-06-03

**Authors:** Chaoliang Wen, Chunning Mai, Ronglang Cai, Qinli Gou, Boxuan Zhang, Junying Li, Congjiao Sun, Ning Yang

**Affiliations:** 1 Department of Animal Genetics, Breeding and Reproduction, College of Animal Science and Technology, China Agricultural University, Beijing, 100193 China; 2 National Engineering Laboratory for Animal Breeding and Key Laboratory of Animal Genetics, Breeding and Reproduction, Ministry of Agriculture and Rural Affairs, China Agricultural University, Beijing, 100193 China

## Abstract

**Background:**

Duration of fertility (DF) is an important economic trait in poultry production because it has a strong effect on chick output. Various criteria or traits to assess DF on individual hens have been reported but they are affected by many nongenetic factors. Thus, a reliable definition and associated genetic parameters are needed. Because egg production is also vital in chicken breeding, knowledge of the relationship between DF and laying performance is needed for designing selection programs.

**Methods:**

We used five traits that consider both fertility and embryonic livability to delineate DF. Phenotypic and genetic analyses were completed for 2094 hens, with measurements of DF at 35 and 60 weeks of age and hatching egg production at 400 days of age (HEP400). The selection differentials for DF and HEP400 were evaluated.

**Results:**

DF is largely independent of the number of oviposited eggs in the peak laying period but both egg production and DF naturally decline with age. The heritability of the five DF traits ranged from 0.11 to 0.13 at 35 weeks of age and increased slightly in the later laying period, ranging from 0.14 to 0.17 (except for efficient duration, time between insemination and the first unhatched egg). Estimates of the genetic correlation for a given trait measured at the two ages were moderate (0.37–0.44), except for efficient duration. However, number of viable embryos depends strongly on egg production. Estimates of genetic correlations of fertility duration day (FDD) at both ages with HEP were weak. Selection for FDD improved DF but without a significant change in laying performance. Selection for increased HEP400 did not contribute to DF improvement.

**Conclusions:**

Although estimates of heritability of the five traits related to DF were low, selection to improve DF based on any one of them is possible. Among these, FDD is an effective selection criterion when the eggs are collected for approximately two weeks after insemination. The best selection procedure for DF improvement would involve multiple measurements at various ages. FDD is independent of laying performance and can be incorporated into a breeding program with egg production to improve reproductive efficiency.

## Background

Chickens are the most ubiquitous of all domesticated animals worldwide. Chicken meat and eggs are not only important sources of high-quality protein for humans [1] but also the most affordable of all livestock products [2]. Genetic selection over the last decades has resulted in a significant improvement in egg and meat production levels [3, 4]. Compared with the intensive selection for production traits, fertility has not received much attention in genetic studies and breeding programs. Fertility is fundamental to reproductive success, so we should expect fertility traits to be under strong selection to maximize reproductive output and minimize the wastage of an animal’s investment in producing gametes. The cost of gametic wastage is likely to be higher for female than male poultry, because females typically invest considerably more into producing yolky ova [5]. In spite of this, fertility of females has received comparatively less attention than that of males within poultry production.

Fertility varies remarkably between breeds, strains, and individuals and is affected by both genetic and nongenetic factors. Except for sperm quality of the male, several physiological factors originating from the female can markedly influence egg fertilization [5]. Previous studies have revealed that female birds can store spermatozoa for prolonged periods in the sperm storage tubules [6–9]. The capability of sperm storage tubules to store and gradually release sperm ensures continuous fertilization during the days following natural mating or artificial insemination (AI). As a consequence, females can lay fertilized eggs for periods of variable length. In practice, the trait of economic interest is the level of fertility and the hen’s ability to maintain a high level of fertility over a long period, termed the duration of fertility (DF), which is usually defined as the number of days after mating or AI when hens lay fertilized or hatched eggs [10]. To determine the DF of an individual hen, early studies usually used pedigreed eggs collected for 18–21 days, beginning on the second day after insemination [11, 12]. The criteria for assessing DF include the efficient (ED, number of days post-insemination until first infertile egg) and maximum duration (MD, number of days post-insemination until last fertile egg) and the number of fertilized eggs or hatched chicks during the observation period [12, 13].

Distinct individual differences in measures of DF have been observed, and all of the DF variables vary in length from a few days to several weeks [6, 12, 14, 15]. The criteria used to assess DF have been estimated to be lowly to moderately heritable [6, 10, 16], indicating some potential for genetic improvement of DF by selective breeding. Due to low fertility in the last few days after AI, the period over which DF measurements are performed can be reduced by several days without leading to significant differences in the ranking of females [12]. Tai et al. [13] suggested that the appropriate selection criterion for DF for ducks was the number of fertilized eggs laid from 2 to 15 days after a single AI with pooled semen. Subsequently, a series of selection experiments in ducks demonstrated the feasibility of selection for this criterion [17–19].

However, several studies have revealed that genetic improvement of DF is accompanied by an increase in embryonic mortality [11, 12]. The number of eggs that are capable of producing a viable embryo depends on their ability for fertilization and their capacity to develop a normal embryo. However, distinguishing embryo mortality from infertility is difficult if mortality occurs in the very early stages of development [20], especially for commercial production. Considering that the main goal of selection to increase DF is to improve the number of hatched eggs, a trait that considers both fertility and embryonic livability may be more suitable for characterizing DF. Brillard et al. [21] conducted divergent selection for increased and decreased numbers of hatched chicks and observed large differences in DF and number of viable embryos per hen between the two lines in generation 5.

Although previous experiments showed that genetic improvement of DF is possible, it has not yet been used on a large scale in commercial breeding programs. A major reason for this is that DF is not well defined, with values that depend on the definition of the trait [6, 12, 22, 23] and DF is affected by many nongenetic factors, such as mating systems [24], the number of spermatozoa inseminated [25], and hen age at data collection [25, 26].

Egg production is also important for reproduction efficiency in poultry production. However, the genetic relationships between DF and laying performance remains to be established. Accurate phenotypic measurements and reliable genetic parameters are vital for choosing a selection criterion and enabling breeders to evaluate the potential impact that selection for DF will have. Thus, the purpose of our study was to determine which DF measurement, considering both fertility and embryonic survival, is preferred for use in chicken breeding programs. Our study also focused on estimating the genetic parameters of DF at various ages and ascertaining the genetic relationship between DF and laying performance.

## Methods

### Animals and management

We used a yellow-feather chicken breed from Guangdong Wen’s Nanfang Poultry Breeding Co., Ltd. (Xinxing, China). This population is a purebred dam line and has been selected for laying performance over 25 years. This experiment included 2094 pedigreed hens from two consecutive hatches (1062 and 1032 chickens from hatches 1 and 2, respectively), which were separated by 12 days. The population of 2094 hens produced from 70 sires and 569 dams. All birds were placed individually in layered cages in one poultry facility and accurately identified by bar codes and cage numbers. The environmental conditions were controlled for ventilation, temperature (19 ~ 28 °C) and photoperiod (16 h light/8 h dark).

### Design of the fertilization experiment

Given that age has an adverse effect on the reproductive success of birds [25, 26], the fertilization experiment was performed during two periods of the hen’s reproductive cycle (at 35 and 60 weeks of age). The phenotypes obtained from the first period provide a reference for early selection. The genetic determination of DF at later production periods could increase the accuracy of selection and thus achieve maximum genetic progress in multistage selection. In the present study, we chose AI as the reproductive method because females were the focus of interest. AI is an efficient technique used by poultry producers to improve reproductive efficiency [24, 27]. Another advantage of AI is the efficient use of males by reducing the number of roosters needed and allowing greater selection pressure on male traits. At each age, AI was performed with duplicate doses on two successive days to eliminate missed inseminations. Specifically, all hens were intravaginally inseminated once per day with 50 µL of diluted semen, which consisted of pooled ejaculates from seven roosters and was diluted 1:1 with a glutamate-based diluent [28].

The effect of sperm quality on DF has been clearly demonstrated by Brillard et al. [25]. To circumvent any undesirable effects of differences in semen quality on subsequent fertility [29], the spermatozoa concentration and motility for each male were assessed with a light microscope. Only males with 3 to 5 billion sperm per milliliter of semen were used for this insemination experiment. Inseminations on two consecutive days were performed in the afternoon by the same technician and were carried out within 30 min of semen collection. The insemination dose was 100×10^6^ viable spermatozoa per AI dose, which is higher than recommended in practice [27] and should fill the sperm storage tubules completely. Hens whose oviducts could not be everted during insemination were eliminated from subsequent analyses.

From a physiological point of view, pedigreed eggs should be assessed for more than 18 days after insemination [10, 11], especially for MD, which is defined as the number of days between the insemination or mating and the final fertile egg or viable embryo [22]. Poultry production is wholly dependent on the supply of day-old chicks at all scales of operation. When hens are at optimal conditions for greater peak and persistency of egg production, the first few eggs after insemination largely determine the fertility and hatchability of eggs set, which has a strong effect on chick output. Thus, we focused mainly on the earliest days after insemination in commercial practice. In addition, to reduce the impact of the fertilization experiment on the supply of hatching eggs, we only collected eggs from days 1 to 15 following the second insemination. Eggs were collected daily and identified individually by marked collection date and hen cage number on the blunt end of the eggshell.

All eggs were classified as normal (settable eggs), cracked, soft-shell, double-yolk or misshapen. Since eggs stored more than eight days have reduced hatchability [30], settable eggs were stored at 12 ± 0.5 °C and moved to incubation within one week under standard conditions. The eggs collected from the last eight days were incubated together. Cracked and soft-shell eggs were not placed in hatcheries because enhanced water loss increases mortality and subsequent dehydration of the embryos, thus decreasing hatching success [31]. Cracked eggs also have a higher risk of bacterial contamination, which may increase the likelihood of contamination of the incubator due to putrefaction, leading to embryonic death [32]. Because it is difficult and inconvenient to distinguish hatched chicks, the settable eggs were examined for the presence of viable embryos by candling on day 18 of incubation [11] and the collection date and cage number of each unhatched egg (containing infertile eggs and dead embryos) were recorded. All information, including the date of collection and the hen’s cage number for the settable and unhatched eggs, were used for subsequent quantification of DF measurements, using a customized R script.

### Measurement of DF traits and laying performance

Hatchability was calculated as the ratio of the number of viable embryos to the number of eggs incubated. Infertile eggs and dead embryos were considered unhatched eggs. We used five traits to assess DF: (1) MD, which is defined as the number of days between AI and the final viable embryo; (2) ED, which is defined as the number of days between AI and the last viable embryo before the first unhatched egg [22]; MD and ED are measures that are commonly used for evaluating DF, because they reflect duration of sperm storage and uninterrupted hatchability, respectively; (3) EN, which is the number of viable embryos during the observation period [12], which determines the chick output, and is an important characteristic in commercial production. Because the first few eggs after AI are more important in commercial practice and MD is too strict, two other criteria were also used to assess DF, i.e. (4) TD, which is the number of days from the day after AI to the last viable embryo before two consecutive unhatched eggs [23]; and (5) FDD, which is fertility duration day and is defined as the number of days from the day after AI to the last viable embryo before two cumulative unhatched eggs, minus any unhatched eggs (0 or 1) before two cumulative unhatched eggs [6]. These five DF traits were calculated for each hen using a customized R script. Only hens with at least five settable eggs collected over a 15-day period were included in the analysis.

For each bird, hatching egg production (HEP) was recorded daily with a barcode scanner until 400 days of age. The age at first egg (AFE) and total HEP at 400 days of age (HEP400) were determined. Mortality was recorded daily. The hen-day laying rate was calculated as the ratio of the number of eggs produced per day to the number of birds in the population that day, expressed as a percentage. Dead hens (N = 53), which were excluded from the calculation of HEP400, were included in the analysis of DF and AFE.

### Statistical analyses

The DF traits measured at the two ages were viewed as different phenotypes in our study. Normality for all traits was assessed using the Shapiro–Wilk test [33] with the Shapiro.test function in the R program (ver 4.0.2). The Wilcoxon rank-sum test was performed to determine differences in DF traits between the two chicken hatches. The difference between measurements of the same trait at the two ages was also investigated using the pairwise Wilcoxon rank-sum test. Differences were considered significant when the *P* value was less than 0.05.

### Estimation of genetic parameters

Pedigree information from six generations was included in the genetic relationship matrix. Heritabilities and phenotypic and genetic correlations were estimated using the AI-REML of DMU software [34] combined with the EM algorithm. The model used for each trait was:

$${y}_{ij}=\mu +{h}_{i}+{a}_{j}+{e}_{ij},$$

where ${y}_{ij}$ is the record of individual $j$ from hatch $i$; ${h}_{i}$ is the fixed effect of hatch (two levels); ${a}_{j}$ represents the random additive genetic effect of chicken $j$; and ${e}_{ij}$ is the residual random error. Heritability was estimated based on a single trait model. Phenotypic and genetic correlations were estimated using a bivariate model.

Improved criteria to select for DF were derived as the averages of the estimated breeding values (EBV) of a given trait at the two ages, after normalizing each EBV to have a zero mean and a variance of 1. All the hens were successively ranked by their average EBV for the various DF traits and the top 50 or 30% of individuals for each of the five traits were selected, resulting in five selected groups. For each group, the selection differential for hatchability was quantified by the difference of the population mean and the mean of the selected hen.

To better understand the relationship between DF and laying performance, the population was separated into two equally-sized groups (the highest and lowest 50% of hens, respectively) based on their EBV of HEP during the observation period or the average EBV for DF. Statistical comparisons of DF variables were then performed between the highest and lowest HEP group with the Wilcoxon rank-sum test. In addition, statistical comparisons of HEP400 and AFE were then performed between the highest and lowest DF group with one-way analysis of variance.

## Results

### Duration of fertility after insemination

**Fig. 1:**
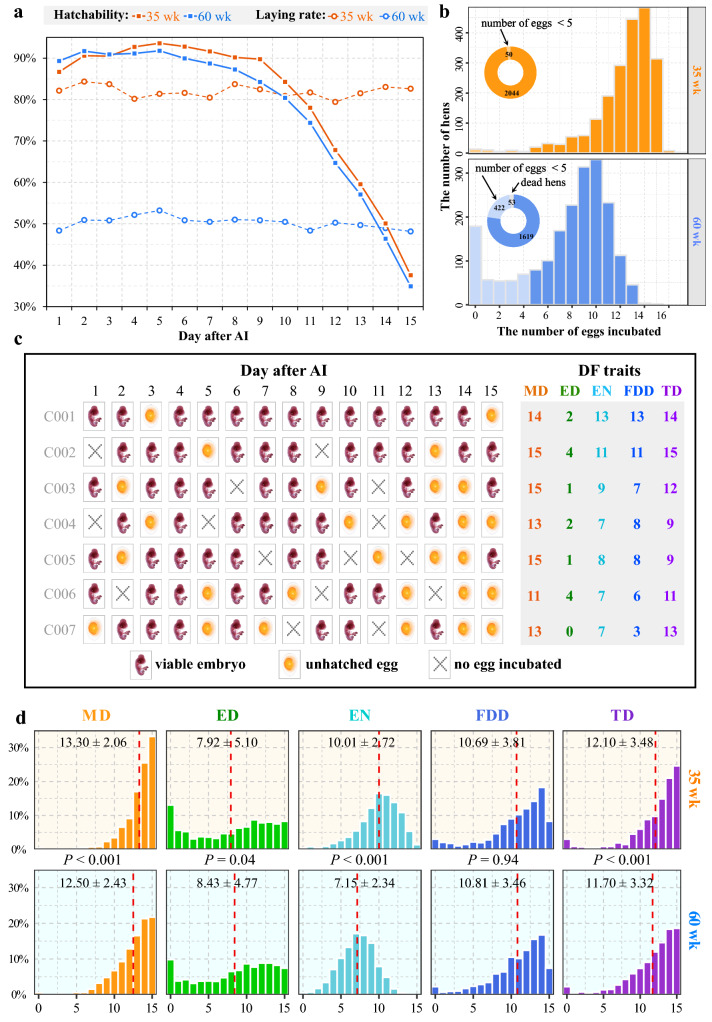
Duration of fertility (DF) and the five traits to measure it. **a** Hen-day laying rate and hatchability at 35 and 60 weeks of age. **b** Histogram of the number of eggs incubated for each hen at 35 and 60 weeks of age. **c** Examples of five traits used to determine DF; and (**d**) Histogram of the five DF traits at 35 and 60 weeks of age. The red dashed line is the mean value of the corresponding trait at a given age. Data are expressed as the mean ± SD. The estimates may not represent the true statistics for these DF variables since the eggs were only collected during 15 days. The *P* value indicates the significance of the difference between the same variable measured at two ages

Hen-day laying rate and hatchability are shown in Fig. 1a for the two experimental periods. Hen-day laying rate was substantially lower at 60 weeks of age (50.3%) than at 35 weeks of age (82.0%). To reduce the impact of the fertilization experiment on the commercial demands for day-old chicks, eggs were collected only during the first 15 days after insemination. The changes in hatchability with days since insemination were similar at 35 and 60 weeks of age. Specifically, there was an obvious increase in hatchability from day 1 to day 2. After the hatchability peak on day 5, it decreased slightly on day 6 and remained relatively constant until day 8 or 9, after which it dropped linearly and approached 35% at day 15. However, hatchability declined earlier in older females, with hatchability on days 4 to 15 being significantly higher for younger hens than for hens at 60 weeks of age (*P* < 0.01). The higher rate of laying at 35 weeks of age may explain the higher rate of decline in hatchability from days 9 to 15 for the younger flock. To prevent any undesirable effects of the number of eggs incubated on the DF analyses, hens that produced less than five eggs during collection period were excluded from the DF analyses. In total, 2044 and 1619 chickens were used in the calculation of the five DF traits at 35 and 60 weeks of age (Fig. 1b), respectively. Several examples of the evaluation of DF variables are shown in Fig. 1c. Hen age had a considerable effect on the DF traits and selection based on a record at one age is not recommended. Thus, the DF variables measured at the two ages were viewed as different traits, and the average EBV of the DF trait was used for further analyses.

Descriptive statistics for all phenotypes are summarized in Table 1. Some hens laid more than five settable eggs, but all were infertile eggs or dead embryos. Thus, a minimum of 0 was observed for each DF trait. The coefficient of variation (CV) of AFE was less than 5% as a result of artificial lighting management in the coops. However, the records of egg production covered a longer period and variation in HEP400 was high, with a CV value of 15.0%. Remarkably, high phenotypic variation was observed in the DF traits, with CV ranging from 15.5 to 64.4% at 35 weeks of age and from 19.4 to 56.6% at 60 weeks of age. None of the DF traits were significantly different between hatches in the two fertilization experiments. However, the age effect was significant for MD, ED, EN, and TD, but not for FDD, as shown in Fig. 1d. MD, EN, and TD decreased significantly with age, especially EN, dropping from 10.0 to 7.2 (*P* < 0.001). However, ED was significantly higher at 60 weeks of age than at 35 weeks of age (8.4 vs. 7.9, *P* = 0.04), which may be explained by the higher egg production rate of younger hens, resulting in a shorter period for the first unhatched egg after insemination.

**Table 1:** Descriptive statistics of duration of fertility (DF) and laying performance

	Traits	N	Mean	SD	CV (%)	Min.	Max.
DF traits at 35 weeks	MD	2044	13.30	2.06	15.49	0	15
	ED	2044	7.92	5.10	64.35	0	15
	EN	2044	10.01	2.72	27.17	0	15
	FDD	2044	10.69	3.81	35.63	0	15
	TD	2044	12.10	3.48	28.75	0	15
DF traits at 60 weeks	MD	1619	12.50	2.43	19.42	0	15
	ED	1619	8.43	4.77	56.59	0	15
	EN	1619	7.15	2.34	32.77	0	14
	FDD	1619	10.81	3.46	32.03	0	15
	TD	1619	11.70	3.32	28.41	0	15
Laying performances	AFE	2094	158.15	6.83	4.32	136	195
	HEP400	2041	181.88	27.26	14.99	37	235

It should be noted that eggs were collected from day 1 to day 15 following AI, and the estimates of the various DF traits may not represent the real statistics for these characteristics, especially for MD

*MD* maximum duration, *ED* efficient duration, *EN* number of viable embryos, *FDD* fertility duration day, *TD* number of days from the day after AI to the last viable embryo before two consecutive unhatched eggs, *AFE* age at first egg, *HEP400* hatching egg production at 400 days of age, *N* number of non-missing values for each trait, *CV* coefficient of variation

### Genetic parameters of DF traits

None of the DF variables measured in our study exhibited a normal distribution (Shapiro–Wilk test, *P* < 0.001), although the distribution of EN was close to normal. Therefore, the DF traits were regarded as ordinal traits and generalized linear mixed models were used to estimate genetic parameters.

**Fig. 2:**
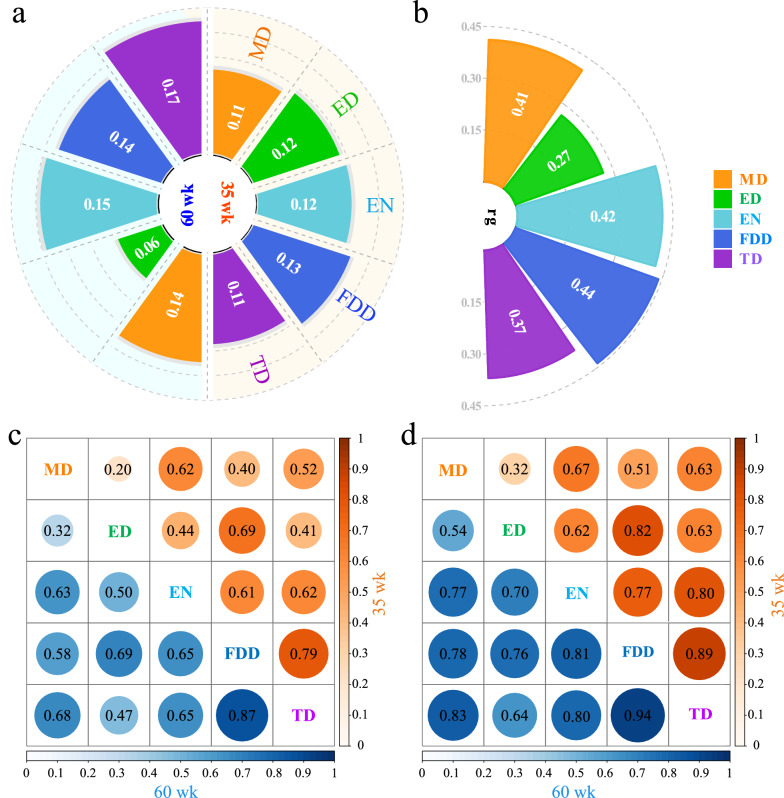
Genetic properties of the five DF traits. **a** Heritability for the five DF traits at 35 and 60 weeks of age; and (**b**) Genetic relationships between the same variable measured at two ages. **c** Phenotypic relationships among the five DF traits at 35 weeks (above the diagonal) and 60 weeks (below the diagonal); **d** Genetic relationships among the five DF traits at 35 weeks (above the diagonal) and 60 weeks (below the diagonal). For panels (**c**) and (**d**), the background color represents the relationship among the five DF traits at the corresponding ages

Estimates of heritability for the five DF traits were similar at 35 weeks of age (0.11–0.13, Fig. 2a). Estimates of heritability for MD, EN, FDD, and TD were slightly higher at 60 weeks of age (0.14–0.17) but lower for ED (0.06). Estimates of genetic correlations for the same trait measured at the two ages were moderate, i.e. 0.41, 0.42, 0.44, and 0.37 for MD, EN, FDD, and TD, respectively, but only 0.27 for ED (Fig. 2b). Phenotypic correlations between the DF variables measured at a given age are shown in Fig. 2c. Except for MD and ED, the DF traits correlated moderately to highly with each other. Phenotypic correlations were slightly higher at 60 weeks of age (0.50–0.87) than at 35 weeks of age (0.40–0.79). Estimates of genetic correlations between the DF traits were very high, ranging from 0.32 to 0.94 (Fig. 2d).

### Comparison of the effect of selection for different DF traits on hatchability

**Fig. 3:**
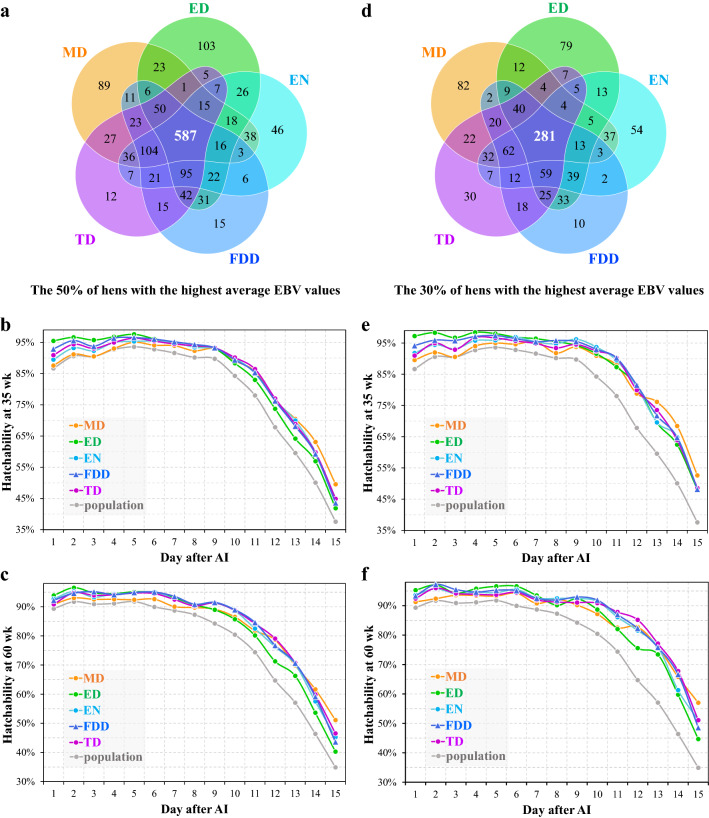
Comparisons of response to selection on the five DF traits. Overlap analyses of the hens shared between the top 50 (**a**) or 30% of hens (**d**) selected according to the average EBV for each of the five DF traits, respectively. The changes in hatchability at 35 or 60 weeks of age of the top 50 (**b**, **c**) or 30% of hens (**e**, **f**) selected based on the average EBV for each the five DF traits

To further determine which trait is the most appropriate for improving DF, all the hens were sorted by their average EBV for each DF trait and the top 50% chickens (N = 1047) for each trait were considered as a group. Only 587 birds (56.1%) were present in the top group for all traits (Fig. 3a). The top groups for MD and ED included 89 (8.5%) and 103 (9.8%) exclusive birds, respectively. These results indicate that selection on different DF traits will result in different genetic progress.

As shown in Fig. 3b, c, selecting on any one of the five DF traits can improve hatchability. Among the five top groups, the MD group exhibited the lowest hatchability during the first eight days and the highest hatchability during the last two to three days. Conversely, the top ED group had the highest hatchability during the first five days but the hatchability of this group declined on day 6 and showed the lowest hatchability from day 9 to day 15. In addition, the heritability estimate for ED was low at 60 weeks of age. Thus, MD and ED may not be the best traits to select on to improve DF. With regard to the three other traits, the top FDD group had higher hatchability during the first nine days than the top TD and EN groups. Similar results were also observed when the 30% of chickens (N = 628) with the highest average EBV for each DF trait were used (Fig. 3d–f).

### Relationship of DF traits with the number of eggs incubated

**Fig. 4:**
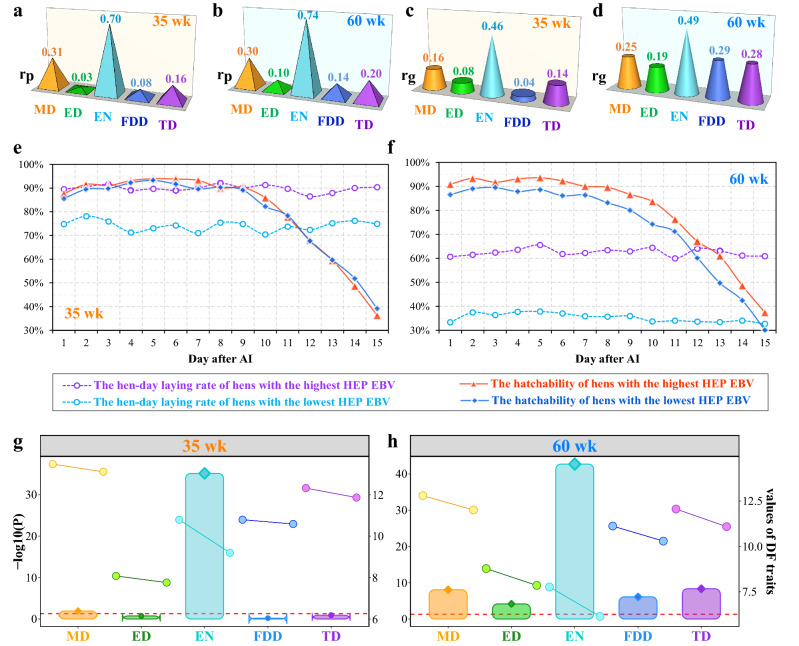
Relationship between DF traits and hatching egg production (HEP) during the collection period. Phenotypic (**a**, **b**) and genetic (**c**, **d**) relationships between number of eggs incubated and the five DF traits at 35 and 60 weeks of age, respectively. Differences in laying rate and hatchability between the highest (N = 1047) and lowest 50% of hens (N = 1047) selected from the EBV of HEP at 35 (**e**) and 60 weeks of age (**f**), respectively. Differences for the five DF traits at 35 (**g**) and 60 weeks of age (**h**), respectively, between the highest and lowest 50% of hens selected based on the EBV of HEP. For panels (**g**) and (**h**), the bar plots show the *P* values for the Wilcoxon rank-sum test between the two groups. The red dashed line shows the significance threshold (*P* = 0.05). The points located to the left and right of the bar indicates the mean value of the hens in the highest and lowest HEP EBV groups, respectively

We also evaluated the associations of each DF trait with HEP during the collection period. The phenotypic relationship of EN with the number of eggs incubated at 35 weeks of age was 0.70 (Fig. 4a). A similar result was found at 60 weeks of age (Fig. 4b), indicating that EN strongly depends on egg production. Phenotypic correlations of MD with number of eggs incubated were equal to 0.31 and 0.30 at 35 and 60 weeks of age, respectively. Phenotypic correlations of HEP with any one of the three other DF traits at the corresponding ages were less than 0.20, especially for FDD and ED, which were largely independent of the number of settable eggs.

Estimates of genetic correlations of each DF trait with HEP are shown in Fig. 4c, d. Moderate correlations were observed between EN and HEP at both ages (0.46–0.49), which further corroborated the idea that EN depends strongly on the actual number of eggs incubated. Estimates of the genetic correlation of HEP with each of the four other DF traits at 35 weeks of age were weak, with values ranging from 0.04 to 0.16 (Fig. 4c). However, estimates of the genetic correlation of HEP with each of the four other DF traits were slightly larger for the later laying period, ranging from 0.19 to 0.29 (Fig. 4d).

To further explore the effect of the number of eggs incubated on DF, the breeder flock was separated into two equally-sized groups based on the EBV for HEP at the corresponding age. Comparisons of DF traits between the highest (N = 1047) and lowest HEP EBV groups (N = 1047) are shown in Fig. 4e–h. At both ages, the hen-day laying rate was clearly greater for the highest HEP group than for the lowest HEP group (Fig. 4e, f). As the oviduct function of an aging flock grows senescent, the DF will naturally decline, which coincides with a decrease in egg production (Fig. 4f). Specifically, as shown in Fig. 4h, the average MD, ED, EN, FDD, and TD were significantly higher for the highest HEP group than for the lowest HEP group at 60 weeks of age (*P* < 0.001). However, there was no obvious distinction in hatchability between the two groups at a younger age (Fig. 4e). These results indicate that DF is largely independent of the actual number of oviposited eggs when the reproductive systems of hens are at optimal conditions. The average ED, FDD, and TD were not significantly different between the two groups at 35 weeks of age (*P* > 0.05, Fig. 4g). However, average MD and EN were higher for the highest HEP group than for the lowest group (*P* < 0.001), especially for EN.

### Genetic relationships of FDD with laying performance

**Fig. 5:**
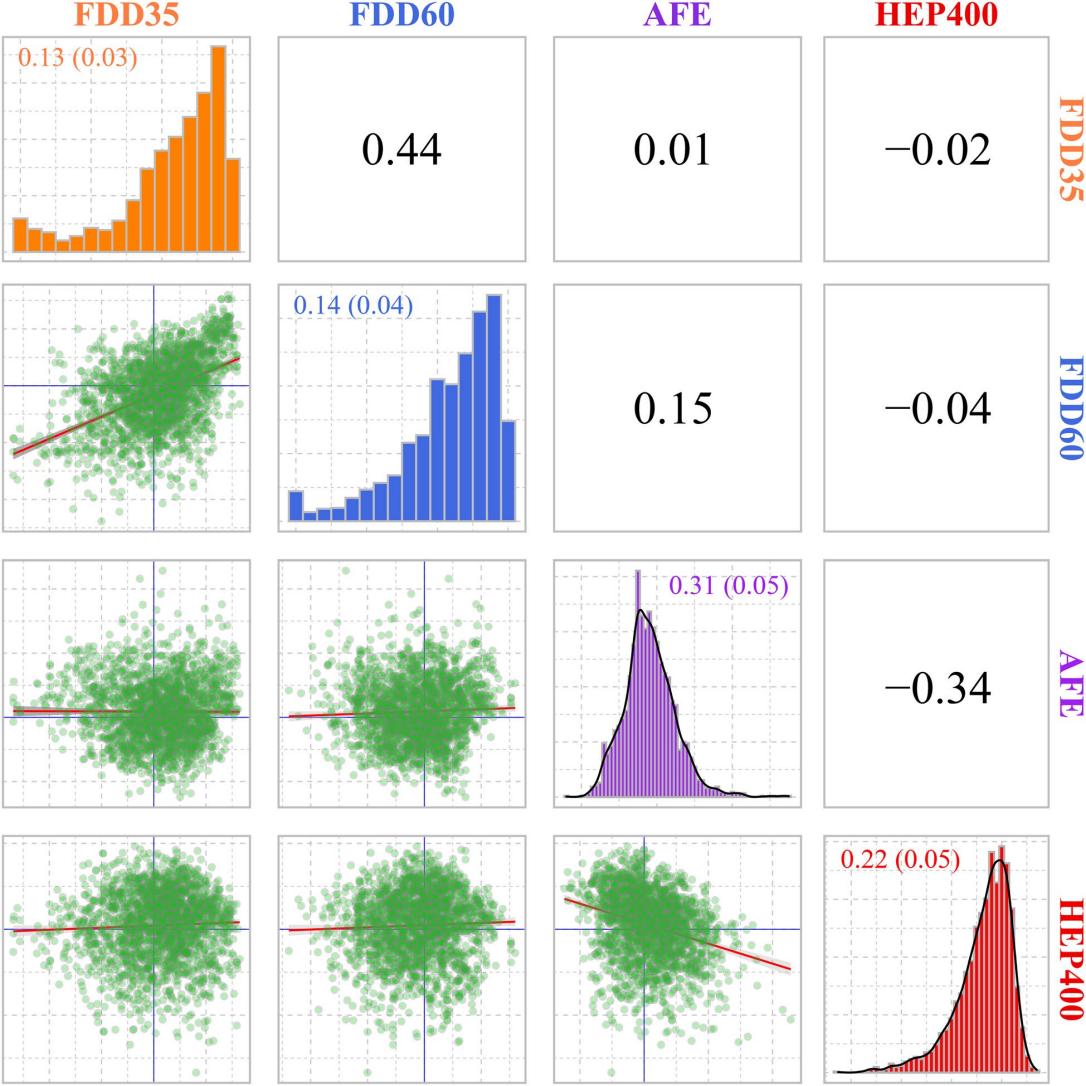
Genetic relationship between fertility duration day (FDD) and laying performance. FDD35 and FDD60 represent FDD measured at 35 and 60 weeks of age, respectively. Estimates of genetic correlations are shown above the diagonal. Scatter plots of the estimated breeding value (EBV) between two traits are shown below the diagonal. The diagonal shows the histogram of each trait. Estimates of heritability and their standard error for each trait are presented on the diagonal

As noted above, FDD is one of the efficient indexes for characterizing DF. Thus, we chose FDD to further assess the relationship between DF and laying performance. Estimates of heritability for AFE and HEP400 were 0.31 and 0.22 (Fig. 5), respectively. HEP400 was moderately and negatively correlated with AFE (− 0.34). However, the genetic correlations between FDD at both ages and HEP400 were negligible, with estimates near 0 (− 0.02 and − 0.04, respectively). The genetic relationship between FDD and AFE was also weak, although it was equal to 0.15 at 35 weeks of age. Generally, no clear genetic association between DF and laying performance was observed.

**Fig. 6:**
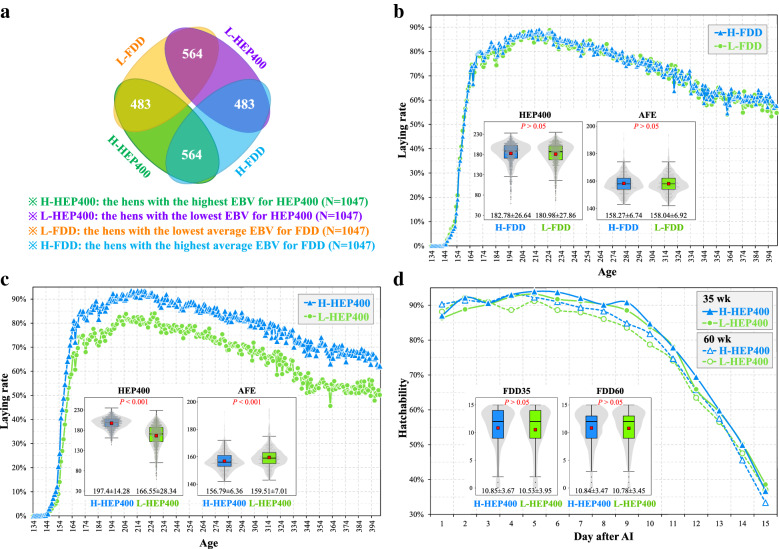
Response to selection for FDD and hatching egg production at 400 days of age (HEP400). **a** Overlap analysis of the hens shared among the birds with the highest and lowest EBV for HEP400 and birds with the highest or lowest average EBV for FDD. **b** Differences in laying performance between the highest and lowest 50% of hens based on the average EBV for FDD. The line chart presents the difference in hen-housed laying rate between the two groups. The boxplots show the differences in HEP400 and age at first egg (AFE) between the two groups. **c** Differences in laying performance between the highest and lowest 50% of hens selected based on EBV for HEP400. The line chart presents the difference in hen-housed laying rate between the two groups. The boxplots show the differences in HEP400 and AFE between the two groups; and (**d**) Differences in DF between the highest and lowest 50% of hens based on EBV for HEP400. The line charts present the difference in hatchability at 35 and 60 weeks of age between the two groups. The boxplots show differences in FDD at 35 and 60 weeks of age between the two groups. For each boxplot, the central red point indicates the mean of the corresponding group and the data are expressed as the means ± SD

To better understand the above result, all the hens were successively ranked by the average EBV for FDD or the EBV for HEP400 and the highest and lowest 50% of birds for each of the traits were selected, resulting in four selected groups. The number of birds (N = 564) that were in both the highest FDD group and the highest HEP400 group was significantly larger than those (N = 483) in both the highest FDD group and the lowest HEP400 group (*P* < 0.05, χ^2^ test, Fig. 6a). The average FDD phenotype was greater in the top FDD group than in the bottom FDD group at both ages (*P* < 0.001). However, the hen-housed laying rate was similar for the highest FDD group and the lowest FDD group (Fig. 6b). There were also no significant differences in HEP400 and AFE between the highest and lowest FDD groups (*P* > 0.05). Comparisons between the highest and lowest HEP400 groups are shown in Fig. 6c, d. The highest and lowest HEP400 birds displayed distinct laying rates (Fig. 6c). The top HEP400 group had higher averages for HEP400 and AFE than the bottom HEP400 group (*P* < 0.001). There was no obvious discrepancy in hatchability between the highest and lowest HEP400 groups at either age (Fig. 6d). Specifically, the average FDD was not significantly different between the top and bottom HEP400 chickens at either age (*P* > 0.05).

## Discussion

Duration of fertility is a trait of major economic importance in poultry production because it has a strong effect on chick output. Increasing DF and thus improving reproductive efficiency would be economically beneficial. The traits used to assess DF in previous studies were generally MD, ED, and the number of fertilized eggs or hatched chicks during the observation period [12–14]. The trait MD is of a cognitive nature, showing the greatest possibility of laying fertilized eggs after a single insemination or mating. The trait ED is considered particularly important because of its practical significance [26]. However, there are some limitations in applying these traits in commercial practice: (1) eggs need to be assessed over a longer period to ensure the accuracy of the measurement [10], especially for MD; (2) the numbers of fertilized eggs and hatched chicks depend strongly on the actual number of oviposited eggs [12, 21]; notably, hens with more settable eggs will have a greater probability of achieving a larger number of fertilized eggs and hatched chicks; and (3) hens with the same number of fertilized eggs or hatched chicks may exhibit a significantly different fertility or hatchability during the first few days after AI or natural mating. The first few eggs largely determine the fertility and hatchability of the eggs set, which are more important in commercial production, especially for farms that use AI.

In the current study, five traits, MD, ED, EN, FDD and TD, were used to characterize DF. The highlights of our study are as follows. First, we adopted a new trait, FDD, to delineate DF, because it is more suitable when the pedigreed eggs are collected for approximately two weeks after insemination. Second, we evaluated the genetic correlation of FDD with egg production and revealed that FDD is independent of laying performance. Selection for egg production, combined with the fertility of those eggs, may be a satisfactory route for improving reproductive efficiency. In addition, to the best of our knowledge, this is the largest-scale analysis of genetic parameters for DF in poultry from the same generation to date. An analysis of an entire population should generate more valid data than a small sample size, since a sample size that is too small would reduce the power of the study and increase the margin of error.

DF is the result of a complex interaction of traits from two individuals with different characteristics, sperm quality for males and oviduct physiology for females. We observed high individual variability in DF at the two ages evaluated, even when insemination conditions were considered optimal. Similar results were also found for ducks [35], geese [36], quails [37], and turkeys [38]. Estimates of heritability of the DF traits were approximately 0.12 for the peak laying period and increased slightly for the later laying period (except for ED), which was in accordance with previous estimates for layer-type hens [10]. However, the estimate of heritability for DF was notably lower than previous estimates for ducks [13], which ranged from 0.29 to 0.38. This difference in heritability may be partly due to species or strain differences but is probably mainly attributable to differences in trait definition, hen age at data collection, and length of the observation period. In our study, approximately 35% of the hens were presumably still laying fertile eggs at 15 days after the second insemination, which indicates that the genetic potential of some of the hens had not yet been realized by the end of the observation period. Experiments over longer periods of time should be conducted to determine whether the measurement of DF can be shortened without impacting genetic parameter estimation. In general, the estimates of heritability indicated the existence of genetic variability for DF and thus that DF can be improved through selection.

Several related studies on selection for DF have been published. Selection over eight generations for fertility of frozen-thawed chicken semen was conducted by Ansah et al. [39]. The DF of frozen-thawed semen improved significantly, with the mean increasing from 1 day in generation 1 to 5 days in generation 8. Brillard et al. [21] found that MD, ED, and EN differed significantly between two chicken lines that were divergently selected for number of hatched chicks. Another study in ducks [17] reported a 12-generation selection experiment with a line selected on the number of fertile eggs after insemination and an unselected line. The average response per generation was 0.40 for number of fertile eggs, 0.45 for MD, and 0.32 for number of hatched ducklings. Moreover, the fertility in the selected line was 92% at day 4 and then decreased slowly to 81% at day 8, while the fertility of the unselected line decreased abruptly at day 4 to 74%. All these results confirm that DF can be improved effectively by genetic selection.

Precise phenotypes are important for genetic selection and a large number of traits has been reported to evaluate individual DF. These traits are highly variable and affected by many nongenetic factors. Thus, a reliable definition of a trait to assess DF and for its application in breeding programs is essential. Coincident with decreasing egg production with age, other indicators of age-related declines emerge, including a decline in fertility. In broiler breeder hens, Brillard et al. [25] observed a statistically significant decline in both ED and MD when inseminations were performed after 55 weeks of age. Gumulka et al. [26] found that the average ED and MD were approximately two days shorter for inseminations of hens at 56 weeks of age compared to 31 and 36 weeks of age. The adverse effect of bird age on DF was also reported for laying hens [12], quails [40], and partridges [41]. Furthermore, the low repeatability of DF traits between ages [12, 39, 42] indicates that genetic selection for DF should be based on multiple measurements at different ages to improve the accuracy of selection for a high DF across ages.

The phenotypic and genetic correlations between the five DF traits examined in the present study were high at both ages (35 and 60 weeks), while MD and ED were not strongly correlated with each other. A similar result was reported by Beaumont et al. [12] in egg-type hens, in which MD was not highly correlated with ED. ED is an important characteristic in commercial production since it reflects uninterrupted hatchability. The relatively lower genetic correlation between ED and MD indicates that MD may not be economically important, which was confirmed by subsequent selection analyses that showed that MD is theoretically associated with the greatest possibility of laying hatch eggs but has a limited effect on DF improvement, especially in the first few days after insemination. The genetic correlation between ED measured at the two ages was low, and the heritability of ED at 60 weeks of age was 0.06. Genetic improvement is the product of the selection intensity applied and the heritability of the trait [43]. Selection based on ED is possible but not efficient. Among the three other DF traits, EN strongly depends on individual egg production. Hens with a higher laying intensity and persistence will lay more eggs, which will, in turn, have a higher probability of achieving complete embryonic development and hatching. As demonstrated here, the average EN was significantly larger for younger hens with a higher laying rate than for younger hens with a lower laying rate. Other reasons for choosing FDD as the selection criterion over TD include its higher genetic correlation at the peak and in later laying periods and its greater selection differential.

In commercial selection programs, chicken breeders must consider a wide array of traits that have some economic importance to achieve the genetic gains needed for commercial success. Persistence of egg production and DF are very important in chicken breeding because these traits are beneficial for chicken reproduction efficiency and are required in both grandparental and parental stocks. Knowledge of the relationship of DF with laying performance is important for optimization of selection programs. It is widely acknowledged that egg-type chickens have a higher laying intensity and persistence than meat-type chickens but the duration of fertility of meat-type chickens [25] is not shorter than that of egg-type chickens [12]. Gowe et al. [44] performed a multiple-trait selection experiment and found that selection for fertility and hatchability had little effect on the genetic improvement in egg production. Here, we estimated the correlation between FDD and egg production and compared the selection differentials for HEP400 and FDD. Our results implied that laying performance and DF are independent traits and that selection for DF or for egg production does not contribute to improvement in the other trait, which is not consistent with the earlier hypothesis of Lamoreux [45] that hens with higher laying rates tend to have a longer DF. Genetic selection for increasing laying intensity and persistence has been highly successful in egg-type poultry. However, there is no evidence that the improvement in egg production has been accompanied by high fertility and hatchability.

## Conclusions

Our results suggest that DF traits that take both fertility and embryonic survival into account have a low heritability in the peak laying period and a slightly higher heritability in the later laying period. The best selection procedure to increase DF includes taking multiple measurements at various ages to improve the accuracy of selection. Genetic selection to improve DF based on MD or ED is possible but may not be efficient as it has a limited effect on DF improvement. With respect to EN, which depends mainly on the actual number of eggs incubated. Another appropriate trait to select for DF in chickens is FDD, which showed a higher selection differential than TD and is independent of laying performance. Thus, selection for FDD has a limited effect on laying performance. Thus, a breeding program including FDD and egg production in a selection index may be more efficient than other programs for commercial chicken production. Our study provides valuable insights into DF traits so that poultry breeders can make informed decisions regarding the optimization of breeding programs for profit maximization.

## Data Availability

The R script used to assess the DF and the datasets generated for the current study are available from the corresponding author on request.

## References

[1] Siegel PB (2014). Evolution of the modern broiler and feed efficiency. Annu Rev Anim Biosci.

[2] Petracci M, Cavani C (2012). Muscle growth and poultry meat quality issues. Nutrients.

[3] Tallentire CW, Leinonen I, Kyriazakis I (2016). Breeding for efficiency in the broiler chicken: a review. Agron Sustain Dev.

[4] Thiruvenkadan AK, Panneerselvam S, Prabakaran R (2010). Layer breeding strategies: an overview. Worlds Poult Sci J.

[5] Assersohn K, Brekke P, Hemmings N (2021). Physiological factors influencing female fertility in birds. R Soc Open Sci.

[6] Wen C, Mai C, Wang B, Li J, Sun C, Yang N (2020). Detrimental effects of excessive fatty acid secretion on female sperm storage in chickens. J Anim Sci Biotechnol.

[7] Holt WV, Fazeli A (2016). Sperm storage in the female reproductive tract. Annu Rev Anim Biosci.

[8] King LM, Brillard JP, Bakst MR, Donoghue AM (1999). Isolation of sperm storage tubules from the uterovaginal junction mucosa of the turkey. Poult Sci.

[9] Yang G, Li S, Zhao Q, Chu J, Zhou B, Fan S (2021). Transcriptomic and metabolomic insights into the variety of sperm storage in oviduct of egg layers. Poult Sci.

[10] Beaumont C (1992). Genetic parameters of the duration of fertility in hens. Can J Anim Sci.

[11] Lodge JR, Fechheimer NS, Jaap RG (1971). The relationship of in vivo sperm storage interval to fertility and embryonic survival in the chicken. Biol Reprod.

[12] Beaumont C, Brillard JP, Millet N, De Reviers M (1992). Comparison of various characteristics of duration of fertility in hens. Br Poult Sci.

[13] Tai C, Poivey JP, Rouvier R (1994). Heritabilities for duration of fertility traits in brown Tsaiya female ducks (*Anas platyrhynchos*) by artificial insemination with pooled muscovy (*Cairina moschata*) semen. Br Poult Sci.

[14] Liu GQ, Zhu JJ, Wang ZY, Jiang XP, Dafalla MM (2008). Analysis of sperm storage ability using duration of fertility in hens. Br Poult Sci.

[15] Adetula AA, Gu L, Nwafor CC, Du X, Zhao S, Li S (2018). Transcriptome sequencing reveals key potential long non-coding RNAs related to duration of fertility trait in the uterovaginal junction of egg-laying hens. Sci Rep.

[16] Brun JM, Mialon MM, Sellier N, Brillard JP, Rouvier R (2012). Inheritance of duration of fertility in female common ducks (*Anas platyrhynchos*) inseminated in pure breeding or in inter-generic crossbreeding with Muscovy drakes (*Cairina moschata*). Animal.

[17] Cheng YS, Rouvier R, Liu HL, Huang SC, Huang YC, Liao CW (2009). Eleven generations of selection for the duration of fertility in the intergeneric crossbreeding of ducks. Genet Sel Evol.

[18] Cheng YS, Rouvier R, Poivey JP, Huang HC, Liu HL, Tai C (2005). Selection responses in duration of fertility and its consequences on hatchability in the intergeneric crossbreeding of ducks. Br Poult Sci.

[19] Cheng YS, Rouvier R, Poivey JP, Tai JJ, Tai C, Huang SC (2002). Selection responses for the number of fertile eggs of the Brown Tsaiya duck (*Anas platyrhynchos*) after a single artificial insemination with pooled Muscovy (*Cairina moschata*) semen. Genet Sel Evol.

[20] Hemmings N, Evans S (2020). Unhatched eggs represent the invisible fraction in two wild bird populations. Biol Lett.

[21] Brillard JP, Beaumont C, Scheller MF (1998). Physiological responses of hens divergently selected on the number of chicks obtained from a single insemination. J Reprod Fertil.

[22] Brillard JP, Antoine H (1990). Storage of sperm in the uterovaginal junction and its incidence on the numbers of spermatozoa present in the perivitelline layer of hens' eggs. Br Poult Sci.

[23] Goerzen PR, Julsrud WL, Robinson FE (1996). Duration of fertility in ad libitum and feed-restricted caged broiler breeders. Poult Sci.

[24] Bernon DE, Siegel PB (1981). Fertility of chickens from lines divergently selected for mating frequency. Poult Sci.

[25] Brillard JP, McDaniel GR, De Reviers M, Drane JW (1989). Expression of several traits of fertility in young and old dwarf broiler breeder hens inseminated with duplicate doses of semen. Poult Sci.

[26] Gumulka M, Kapkowska E (2005). Age effect of broiler breeders on fertility and sperm penetration of the perivitelline layer of the ovum. Anim Reprod Sci.

[27] Mohan J, Sharma SK, Kolluri G, Dhama K (2018). History of artificial insemination in poultry, its components and significance. World Poult Sci J.

[28] Lake PE (1960). Studies on the dilution and storage of fowl semen. J Reprod Fertil.

[29] Parker HM, Karaca AG, Yeatman JB, Frank LR, McDaniel CD (2002). Fertility of broiler breeders following categorization by the OptiBreed sperm quality index when hens are inseminated with a constant number of sperm. Poult Sci.

[30] Abioja MO, Abiona JA, Akinjute OF, Ojoawo HT (2021). Effect of storage duration on egg quality, embryo mortality and hatchability in FUNAAB-a chickens. J Anim Physiol Anim Nutr (Berl).

[31] Kirikci K, Deeming DC, Gunlu A (2004). Effects of egg mass and percentage mass loss during incubation on hatchability of eggs of the rock partridge (*Alectoris graeca*). Br Poult Sci.

[32] Barnett DM, Kumpula BL, Petryk RL, Robinson NA, Renema RA, Robinson FE (2004). Hatchability and early chick growth potential of broiler breeder eggs with hairline cracks. J Appl Poult Res.

[33] Royston JP (1982). An extension of Shapiro and Wilk's w test for normality to large samples. Appl Statist.

[34] Madsen P, Jensen J. A user's guide to DMU. A package for analysing multivariate mixed models. Version 6, release 5.2. Tjele: University of Aarhus. 2013.

[35] Ash WJ (1962). Studies of reproduction in ducks: 1. The duration of fertility and hatchability of White Pekin duck eggs. Poult Sci.

[36] Johnson AS (1954). Artificial insemination and the duration of fertility of geese. Poult Sci.

[37] Reddish JM, Kirby JD, Anthony NB (1996). Analysis of poultry fertility data: 3. Analysis of the duration of fertility in naturally mating Japanese quail. Poult Sci.

[38] Hale EB (1955). Duration of fertility and hatchability following natural matings in Turkeys. Poult Sci.

[39] Ansah GA, Buckland RB (1983). Eight generations of selection for duration of fertility of frozen–thawed semen in the chicken. Poult Sci.

[40] Santos TC, Murakami AE, Oliveira C, Moraes GV, Stefanello C, Carneiro TV (2015). Influence of European quail breeders age on egg quality, incubation, fertility and progeny performance. Braz J Poult Sci.

[41] Mourao JL, Barbosa AC, Outor-Monteiro D, Pinheiro VM (2010). Age affects the laying performance and egg hatchability of red-legged partridges (*Alectoris rufa*) in captivity. Poult Sci.

[42] Liu HC, Huang JF, Lee SR, Liu HL, Hsieh CH, Huang CW (2015). Selection for duration of fertility and mule duck white plumage colour in a synthetic strain of ducks (*Anas platyrhynchos*). Asian-Australas J Anim Sci.

[43] Pollock DL (1999). A geneticist's perspective from within a broiler primary breeder company. Poult Sci.

[44] Gowe RS, Fairfull RW, McMillan I, Schmidt GS (1993). A strategy for maintaining high fertility and hatchability in a multiple-trait egg stock selection program. Poult Sci.

[45] Lamoreux WF (1940). The influence of intensity of egg production upon infertility in the domestic fowl. J Agric Res.

